# Investigating decision rules with a new experimental design: the EXACT paradigm

**DOI:** 10.3389/fnbeh.2015.00288

**Published:** 2015-11-03

**Authors:** Valerio Biscione, Christopher M. Harris

**Affiliations:** ^1^School of Psychology, Plymouth UniversityPlymouth, UK; ^2^Centre for Robotics and Neural Systems, Plymouth UniversityPlymouth, UK

**Keywords:** optimal performance, reward rate, speed-accuracy trade-off, perceptual choice, decision rules

## Abstract

In the decision-making field, it is important to distinguish between the perceptual process (how information is collected) and the decision rule (the strategy governing decision-making). We propose a new paradigm, called EXogenous ACcumulation Task (EXACT) to disentangle these two components. The paradigm consists of showing a horizontal gauge that represents the probability of receiving a reward at time t and increases with time. The participant is asked to press a button when they want to request a reward. Thus, the perceptual mechanism is hard-coded and does not need to be inferred from the data. Based on this paradigm, we compared four decision rules (Bayes Risk, Reward Rate, Reward/Accuracy, and Modified Reward Rate) and found that participants appeared to behave according to the Modified Reward Rate. We propose a new way of analysing the data by using the accuracy of responses, which can only be inferred in classic RT tasks. Our analysis suggests that several experimental findings such as RT distribution and its relationship with experimental conditions, usually deemed to be the result of a rise-to-threshold process, may be simply explained by the effect of the decision rule employed.

## Introduction

Decision-making can be broken down, at least conceptually, into two components: a perceptual process and a decision rule. We define the perceptual process as the mechanism that accumulates information in order to decide between alternative responses. It does not include all the perceptual information an observer is experiencing during a decision/experimental task, but only that which affects the ultimate decision. We define the decision rule as the quantity being optimized during the task, which will therefore establish when enough information has been gathered and a decision can be made. A great amount of work in the decision-making field has focused on the perceptual process implemented in the brain, resulting in several models that can account for a wide variety of data (e.g., LaBerge, 1962; Laming, 1968; Ratcliff, 1978; Busemeyer and Townsend, 1993; Usher and McClelland, 2001; Ratcliff and Smith, 2004; Ratcliff et al., 2004a,b). In most cases, these models assume a noisy accumulation of information toward one of two alternatives, until a decision threshold is reached, whereupon a response is made (e.g., drift diffusion model: Stone, 1960; Laming, 1968; Ratcliff, 1978; Ratcliff and Rouder, 2000; linear ballistic accumulation: Brown and Heathcote, 2008; Ornstein-Uhlenbeck model: Busemeyer and Townsend, 1993). There is, however, a growing interest in the decision rule per se, which affects how participants set their decision thresholds, that is when to stop collecting information and make a decision (Gold and Shadlen, 2002; Bogacz et al., 2006; Holmes and Cohen, 2014; Moran, 2014).

The usual approach to investigating decision rules consists of (1) collecting data from a classic reaction time (RT) experiment; (2) assume a particular perceptual process, usually the drift diffusion model; (3) based on the perceptual process assumed, test different decision rules (for example, see Simen and Cohen, 2009; Bogacz et al., 2010). This approach has serious drawbacks: the predictions of the decision rule depend on the perceptual mechanism assumed, and there are a variety of possible perceptual mechanisms (e.g., Ratcliff et al., 1999; Ratcliff and Smith, 2004; Smith and Ratcliff, 2004). When considering more than two alternatives, there are even more approaches which differ in behavioral and neurobiological assumptions (Krajbich and Rangel, 2011). This leads to a problem when fitting decision rules: which perceptual process should one assume? Moreover, should a decision rule not fit the data, would that be due to the decision rule itself or to the perceptual process assumed? Hypothetically, this issue could be solved by comparing different perceptual processes with different decision rules, but this is rarely done in practice. Even in this case, it is not possible to know if the sample of perceptual processes tested comprises the one used by human participants. This is a serious limitation when the focus of the investigation is on the decision rule, and not the perceptual process. There is also a second limitation of applying perceptual process in testing decision rules: the commonly used perceptual models are employed in case of fast decisions (average RT < 1 s) (Voss et al., 2013) but their applicability for longer decision remain untested, even though real-life decisions may take several seconds.

We believe that there is a need for a new paradigm which will allow researchers in the decision-making field to reduce the interdependence of the perceptual and decision processes. We propose a new paradigm, called EXACT (EXogenous ACcumulation Task) which allows the decision rule to be investigated without the need of making any assumption about the underlying perceptual process.

The perceptual process affects the decision rule by defining a certain relationship between time of response and accuracy of response: *ACC*(*t*), where *t* is the time taken to make a response. *ACC*(*t*) defines the speed-accuracy trade-off: it is the probability of being correct at time *t*, and it is assumed to increase with time so that the slower the response, the more accurate it is (Heitz, 2014). The particular shape of this function depends on the perceptual model assumed. To understand the decision rule without making any assumption about the perceptual process, we need to hard-code *ACC*(*t*) itself into the experimental design. Instead of showing two or more stimuli and having the participant choose the correct one by an endogenous accumulation of information and subsequent increase of *ACC*(*t*), the *ACC*(*t*) can be presented directly to the participants who are asked to make the decision based on this exogenous *ACC*(*t*). We call this paradigm EXACT, because the speed-accuracy trade-off function is presented exogenously to the participant, instead of being an endogenous process. In this task, we are assuming that the perceptual accumulation of information is separable from the decision rule, such that if we replace the perceptual accumulation exogenously, we can observe the decision rule in a meaningful way.

**Figure 1:**
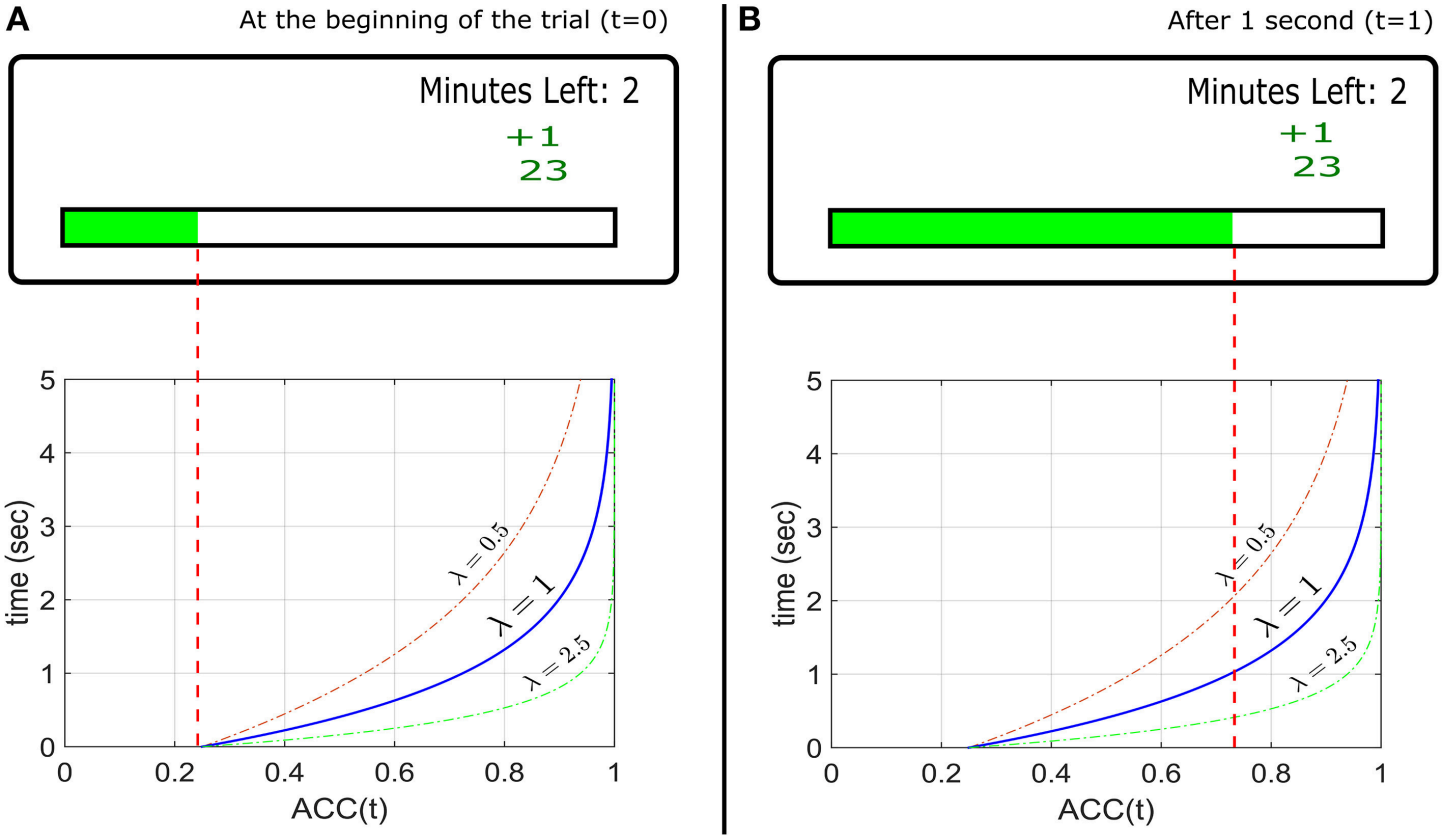
**Top Panels: Example of the experimental design used to implement the EXACT paradigm, showing how the gauge represents the probability at time t assuming a starting point at 25% during a hypothetical trial**. The small green +1 displays the feedback from the previous trial (point won/lost). The number below represents the total current score for that condition. The “minutes left” refers to the minutes left for that condition. **(A)** Top: the screen as seen by the participant at the beginning of the trial (*t* = 0) and **(B)** after 1 s, assuming λ = 1. Bottom: the ACC(t) functions with different λs, representing the probability of getting a reward with time. The gauge on the top panels is only referred to the function with λ = 1. The other functions show how the accuracy would change in time for different λ. For example, with λ = 2.5, after 1 s, the accuracy is equal to ~0.95 (the gauge would be almost completely full).

To present *ACC*(*t*) directly to the participant, a horizontal gauge is displayed. There are no separate visual targets. During a trial, the position of the gauge level is moved to the right corresponding to an increasing *ACC*(*t*) predetermined by the experimenter. Zero probability corresponds to an empty gauge, and unit probability corresponds to a full gauge. At the beginning of the trial the gauge starts at *ACC*(0) (not necessarily zero). The participant wins X reward tokens with probability *ACC*(*t*) when they press a button at time *t*, and loses Y tokens with probability 1−*ACC*(*t*). After the response, a new trial starts. The experimenter can specify a delay between trials. Figure 1 (top) shows an example of the monitor screen shown to the participants. On the bottom panels the corresponding *ACC*(*t*) (an exponential function, as discussed below) is shown (not seen by participants). The axes are inverted so that the horizontal axis corresponds to the direction of the gauge movement.

For an illustrative experiment with the EXACT paradigm, we chose an exponential *ACC*(*t*):

(1) $$\begin{array}{l} ACC\left(t\right)=1+\left(\alpha -1\right)\exp\left(-\lambda t\right) \end{array}$$

so that:

(2) $$\begin{array}{l} t\left(ACC\right)=-\ln\;\left(\frac{\left(ACC-1\right)}{\alpha -1}\right)∕\lambda \end{array}$$

where λ is a parameter controlling how fast the function grows, and α is the value at *t* = 0. In the EXACT paradigm, λ corresponds to the speed of the gauge, and α is the gauge level at the beginning of the trial, that is *ACC*(0). This choice of *ACC*(*t*) was based on ease of mathematical tractability, capability of capturing different aspect of the task, and similarity to the *ACC*(*t*) produced by widely used models^1^. However, other functions could be used to test decision rules. The main point is that the function is completely known by the researcher and not derived from an assumed perceptual process.

The EXACT paradigm can be easily compared with a classic RT task. The λ parameter controls the speed of the gauge, which can be compared to manipulating the trial difficulty (for example, by changing stimulus intensity, stimulus contrast, etc…). When the task is difficult, the rate of accumulation of evidence is smaller, and *ACC*(*t*) grows slower. Similarly, changing α, the gauge level at the beginning of the trial corresponds to the number of alternatives: α = 0.5 represents a 2AFC, α = 0.25 a 4AFC. With the EXACT paradigm it is also possible to explore more complicated designs, such as α = 0.75, which corresponds to a task with three correct alternatives out of four possible choices. Conceptual differences from the classic RT task and limitation of the EXACT paradigm are addressed in the discussion (Limitations of the EXACT paradigm).

By knowing the shape of *ACC*(*t*), it is straightforward to test possible decision rules. Four decision rules have been proposed frequently in the literature (Bogacz et al., 2006; Simen and Cohen, 2009; Harris et al., 2014; see Materials and Methods for further details):

***Bayes Risk (BR*)**. This rule was first used by Wald and Wolfowitz (1948) in proving the optimality for the Sequential Probability Ratio Test. It assumes that decision makers seek to minimize a cost function that is the weighted sum of the time and the error rate, without taking into account the total length of the trial. The optimum response time depend on the ratio between the subjective weights of time and error rate.

***Reward Rate (RR)***. It is defined as the proportion of correct trials divided by the average duration between trials (Bogacz et al., 2006). According to RR, responses should not depend on the payoff matrix used in the task.

***Reward/Accuracy (RA*)**. This has been proposed by Bogacz et al. (2006) by formalizing the COBRA theory of Maddox and Bohil, 1998). It is a weighted difference of RR and accuracy, in which the optimum response depends on the punishment/reward ratio and on the total length of the trial.

***Modified Reward Rate (*RR**_**m**_*)*. This rule has been proposed by Bogacz et al. (2006) and Harris et al. (2014). It describes a situation where a correct response corresponds to a subjective reward, and an incorrect response to a subjective punishment, and participants are trying to maximize the rate of gain (Harris et al., 2014). The optimum response does not depend on the absolute values of punishment and reward, but only on their ratio. RR only takes into account the correct responses (a better definition for RR would be Success Rate, but we use RR to be consistent with the literature), whereas RR_m_ considers both correct and incorrect response and their relative weight. Furthermore, RR_m_ is the optimal strategy in paradigm with fixed task duration (see Materials and Methods; e.g., Simen and Cohen, 2009; Bogacz et al., 2010). Harris et al. (2014) have hypothesized that the RR_m_ may also account for the observations that response times have a reciprocal normal distribution (Carpenter, 1981). As it will turn out, the distributions resulting from the EXACT paradigm, with the current design, does not appear to be reciprocally normally distributed, so this point will be considered more deeply in the discussion.

The purpose of this study was threefold. First, we were interested to see how participants responded to this novel paradigm. In particular, we explored whether participants would choose a response time RT and a response gauge position *ACC*(*RT*) that depended systematically on our manipulation of the *ACC*(*t*) parameters α and λ. Second, we asked whether the data could allow us to distinguish among the four decision rule models. Third, we use the results from the paradigm to explain patterns of behavior that have usually been explained with a rise-to-threshold accumulator process.

## Materials and methods

At the beginning of each trial, a gauge was shown on a monitor screen (Figure 1). The gauge started at a predetermined level and “filled up” according to *ACC*(*t*) (Equation 1). The participant pressed the CTRL key to request a reward. Reward and punishment were in form of *points*. When the participant requested a reward, 1 point was awarded with probability given by the level of the gauge (i.e., *ACC*(*t*)), or 1 point was taken away with probability 1−*ACC*(*t*) (punishment/reward ratio *q* = 1). After every response, a text saying “Please Wait” appeared for 0.5 s (delay *d*). When a reward was awarded, “+1” in green appeared on the screen, and “–1” in red appeared for a loss (Figure 1). Each participant started with 25 points at the beginning of each condition. During each condition, the screen also displayed how many minutes were left as well as the current total accrued points. Each condition lasted 3 minutes regardless of performance (no participant lost all their points), so that faster responses would lead to more trials (and possibly more profit). Participants were instructed to win as many points as possible. They were informed on the relationship between the gauge level and the probability of getting/losing a point, and were given a practice session.

This study was approved by the Faculty of Health and Human Sciences Ethics Committee at Plymouth University, with written informed consent from all participants. All participants gave written informed consent in accordance with the Declaration of Helsinki.

### Procedure

We recruited 17 female and 3 male Psychology students to take part in the study. To increase motivation, they were informed that the participant who won the most points would receive a £10 Amazon voucher. Each participant underwent 2 × 4 conditions. The starting point of the gauge was set to either α = 0.25, or 0.75, and the speed of the gauge was set to λ = 0.166, 0.33, 1, or 2. This means that at the very beginning of the trial the chance of receiving a reward was equal to α and the chance of receiving a punishment was equal to 1−α. After that, the chance of receiving a reward increased according to the *ACC*(*t*) (the chance of getting a punishment decreased accordingly). Higher values of λ indicates faster increase. Sequences were randomized across participants, except that each participant was tested on all the speed conditions for a particular starting point, before being tested on the other starting point condition. The participants performed an initial training session to make sure they understood the task (α = 0.5, λ = 1). They were then given another training session at the beginning of each starting point condition (in the training we set α equal to the starting point for that condition and λ = 1). They were allowed to take a break between conditions. There was no response deadline.

### Decision rules predictions

Each decision rule generates different predictions based on the assumed *ACC*(*t*). The mathematical formulation of the four decision rules is similar to the one in Bogacz et al. (2006), with few minor differences: firstly, they use the parameter “Error Rate” (*ER*(*t*)) instead of *ACC*(*t*). The parameterization in terms of *ACC*(*t*) was found to be more convenient for the experimental design employed here (*ER*(*t*) = 1−*ACC*(*t*)). Conversely to Bogacz et al., the additional penalty delay following an error was ignored, since it is not used in our experiment and it is not common in classic RT paradigms. We also did not separate decision time (the time in which the information is accumulated and therefore accuracy increases) from non-decision time (sensory and motor processing), because in the EXACT paradigm any time spent on the trial increases accuracy.

The mathematical formulation of the decision rules with the corresponding optimum response time t^*^ is shown in Table 1. In these formulae *d* is the delay across trials, *t* is the time from the beginning of the trial to the participant's response, *q* is the weight of accuracy relative to the speed of reward (Bogacz et al., 2006) and it is assumed to be subjective and dependent on individual differences.

**Table 1:** Summary of decision rules with associated optimum response time t^*^ for the exponential *ACC*(*t*) in Equation (1).

**Decision rule**	Optimum response (t^*^) by assuming *ACC*(*t*) = 1+(α−1)*exp*(−*λt*)
*BR* = −[*t*+*q*(1−*ACC*(*t*))]	$t_{BR}^{*}=-\frac{\ln\;\left(\frac{1}{\lambda q\left(1-\alpha \right)}\right)}{\lambda }$
*RR* = *ACC*(*t*)∕(*t*+*d*)	$t_{RR}^{*}=-d-\frac{W_{-1}\left(\frac{\exp\left(-\lambda d-1\right)}{\alpha -1}\right)+1}{\lambda }$
$RA=RR-\frac{q}{d}\left(1-ACC\left(t\right)\right)$	No explicit form
$RR_{m}=\frac{ACC\left(t\right)-q\left(1-ACC\left(t\right)\right)}{t+d}$	$t_{RR_{m}}^{*}=-d-\frac{W_{-1}\left(\frac{\exp\left(-\lambda d-1\right)\;}{\left(\alpha -1\right)\left(q+1\right)}\right)+1}{\lambda }$

**Figure 2:**
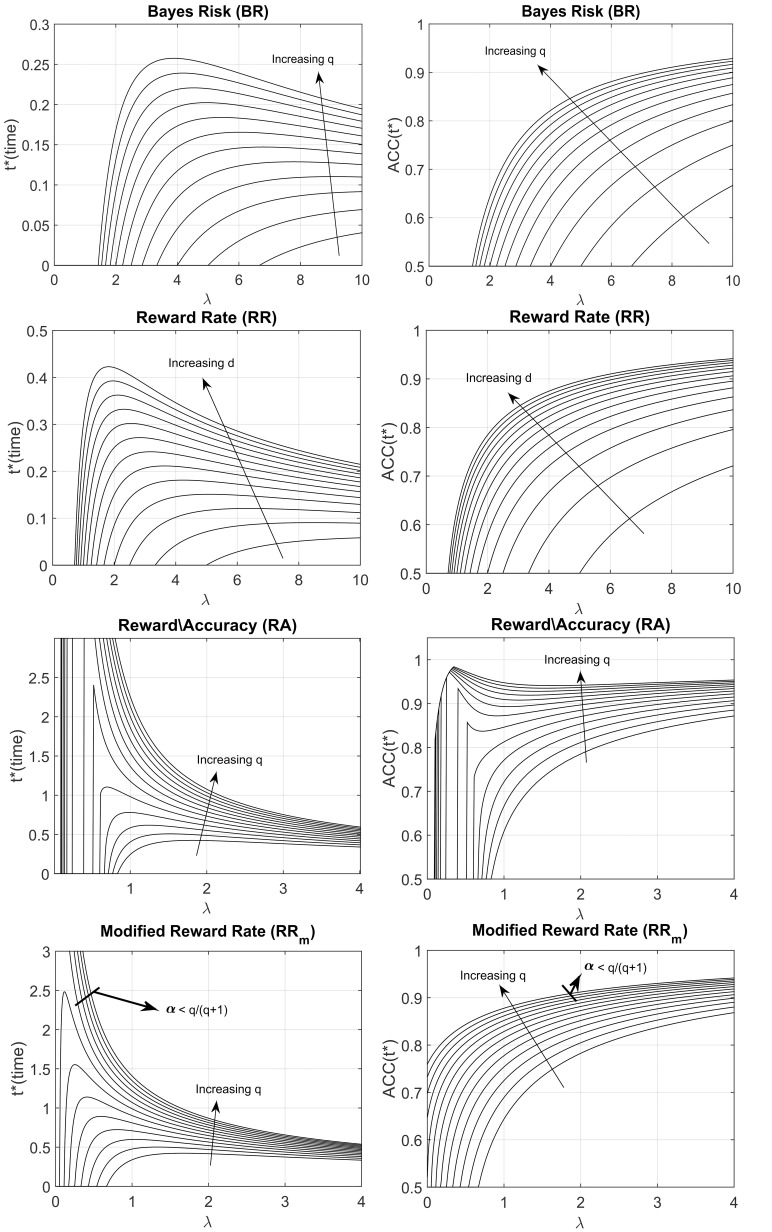
Optimal response time, t* (left column) and ACC(t*) (right column) vs. λ for the four decision rules, with different parameters q and d. *ACC*(t*) indicates the optimum level of accuracy that has to be reached upon responding. The arrows indicate how the shape changes when the d or q parameter is increased. The q parameter represents the subjective punishment/reward ratio; the d parameter represents the length of the trial, excluding the reaction time. Increasing q or increasing d corresponds to an increase in the optimum response time (t*) and a consequent increase of ACC(t*). For the Modified Reward Rate (**bottom panels**), two very different t* shapes are produced with different combinations of q and α (the starting point of the gauge).

We derived the optimum time of responding t^*^ (right column of Table 1) by setting the derivative of the decision rule to 0 and solve for *t* (see Appendix in Supplementary Materials for details). Figure 2 shows both the optimum t^*^ as a function of λ for different parameters value (how long a participant should wait before responding, left panel), and the *ACC*(t^*^) value (what accuracy level is reached upon responding, right panel), assuming the exponential *ACC*(*t*) in Equation (1). For most models, the optimum response t^*^ goes to 0 when λ goes to 0, which means that when the accumulation of evidence in time is too slow (because the trial is difficult, the stimulus is dim, etc…), then it is not convenient to spend long time accumulating information. This is not true for the RR_m_ model with some combination of parameters α and *q*. In particular, the optimum t^*^ goes to infinity when λ goes to 0 given that $\alpha <\frac{q}{q+1}$ (see Figure 2, bottom panels, and Appendix in Supplementary Materials for derivation) and goes to 0 when $\alpha <\frac{q}{q+1}$. The RR_m_ model is particularly relevant because it is the only criterion that actually maximizes the gain for a task (including but not limited to the EXACT paradigm) with a fixed task duration (in subjective utility value, see Harris et al., 2014)^2^. For RR_m_ the parameter *q* can be interpreted as the punishment/reward ratio. The experimenter can try to affect this parameter (by using monetary reward, sound feedback, instruction set, etc…), but other characteristics may be more relevant for the participants, such as intrinsic loss avoidance.

### Statistical analysis

Due to small sample size (sometime less than 15 responses) obtained in some conditions (especially with small λ), we aggregated the data by calculating the median response for each participant. We fitted the three decision rules (BR, RA, RR_m_) response predictions (the condition-dependent t^*^ shown on the right side of Table 1) to the aggregated median responses by an optimization procedure that minimizes the least squares (MATLAB *fminsearch*). RR did not need to be fitted because it is parameter free (*d* is assumed to be known by the participant to be equal to 0.5). For BR, RA and RR_m_, the parameter *q* was estimated with two different approaches: by leaving *q* free to vary across the two α conditions or by fixing it to be equal for the two α conditions. The value of *q* was bounded to be positive.

To analyse how RT and *ACC*(*RT*) changed within a particular condition, the responses were binned for each participant and each condition according to the time elapsed from the beginning of the trial. This was made in order to have the same dataset size for each participant, regardless of the number of responses produced. For the λ = 0.166 and λ = 0.33, bins of 10 s were used and for λ = 1 and λ = 2, bins of 5 s were used. The difference in bins was due to generally slower RT with smaller λ in the α = 0.25 condition, and longer bins were needed to include enough datapoints for reliable statistics. The mean and standard deviation were calculated for each bin, resulting in two time series for each participant and each condition. We computed two linear regressions (mean against time and standard deviation against time) for each dataset, and the resulting slopes were analyzed with a Two-way repeated measure ANOVA and a multiple comparison test.

To understand the sequential dependency along trials, the average partial autocorrelation function (PACF) for each participant and each condition was computed. This function returns the autocorrelation between a response *RT*_*t*_ and *RT*_*t*+*lag*_ removing the dependency through *RT*_*t*+1_ and *RT*_*t*+*lag*−1_. We used a maximum lag of 10. The average PACF across all participants was computed by averaging the individual PACF values for each condition.

Participants' RTs were grouped in order to have a single distribution for each condition by using the Vincentizing method (Ratcliff, 1979). To calculate the response in the rate domain, we computed the rate for each response (1/RT), then standardized the data into z-scores for each participant and each condition, which allowed us to collapse all the data together (Harris et al., 2014).

Any *ACC*(*RT*) distribution must be truncated because *ACC*(*RT*) is bound to lie between 0.25 or 0.75 (depending on the α condition) and 1. We wanted to check whether the *ACC*(*RT*) distribution generated by each participant was close to a truncated Normal. This was complicated by small individual samples, and by the fact that aggregating truncated Normal with different moments makes it ambiguous whether the original distributions were truncated Normal or not. We excluded the conditions where truncation was severe, that is when the sum of mean and two standard deviations was over one and the difference between mean and two standard deviations below α (the truncation point). The value of two for the standard deviation was chosen as a balance between excluding truncated datasets and not excluding too many data points (see Harris et al., 2014). The remaining distributions were standardized into z-scores and collapsed together. By excluding the most severely truncated distribution and aggregating the remaining standardized distributions, we expect to obtain a normal distribution with a milder truncation, if the original distributions were truncated Normal.

## Results

**Figure 3:**
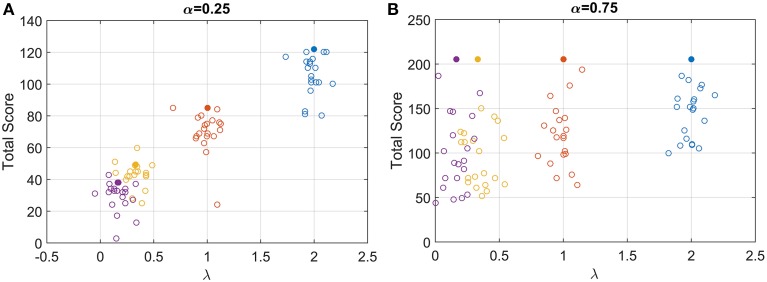
**Open circles: total score for each participant for the low (A) and high (B) starting point condition**. For clarity, each circle is plotted with a random horizontal offset. **Filled circles:** expected score with optimum performance for the four speed conditions and the two starting points conditions.

The examination of the total score gained (points at the end of each condition) can be used to understand how participants responded to the task. Their score can be compared to the optimum average score (expected amount of point at the end of each condition, given that participants were actually trying to maximize the amount of point earned). The optimum expected score was computed by finding the maximum points that could be won for a fixed task duration (RR_m_ with *q* = 1 and *d* = 0.5) (Figure 3, solid circles). For α = 0.25, the total score increased systematically with λ (Figure 3A, open circles), and clearly demonstrates that participants were sensitive to the speed of the gauge. As the gauge level increased it was possible to obtain more points in the available 3 min, and although there was variability among participants, some were close to the optimum performance.

For α = 0.75, the optimum response is t^*^ = 0 for all values of λ, and the maximum score was always 205 points (filled circle in Figure 3B). Participants scores were more variable than before and depended on λ, with an increasing mean and decreasing variance with λ. Even though participants could not possibly reach a *RT* = 0, they should have had the same average RT for the four λ conditions when α = 0.75, if they were maximizing RR_m_ with *q* = 1 and *d* = 0.5.

**Figure 4:**
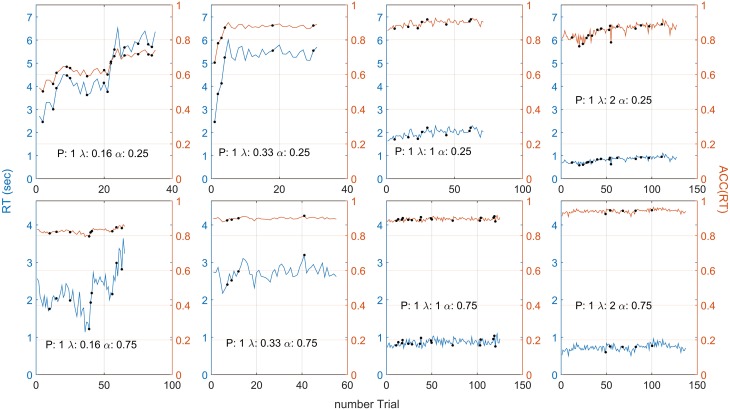
**Participant 1 responses in terms of RT (blue lines) and *ACC*(*RT*) (orange lines) for each λ and α condition**. The black dot indicates that a punishment has been given on that trial. On average, *ACC*(*RT*) increases with λ and is usually lower for the α = 0.25 condition. Note that the different number of trials depended on the condition (which always lasted 3 minutes). For example, when α = 0.25, λ = 0.16, the gauge grew more slowly, participant's RT were slower, and less trials were made. Note that high variability in RT does not always correspond to high variability in *ACC*(*RT*). Compare this plot with Figures 5A,B, for the grouped RT and *ACC*(*RT*) across participants. Note that the two α conditions have two different scales.

Figure 4 shows responses for one representative participant in terms of RT (blue lines) and positional responses along the gauge, *ACC*(*RT*) (orange lines). The positional response *ACC*(*RT*) were variable but typically showed an increasing trend with higher λ. Positional responses were lower for α = 0.25 than 0.75. This participant was selected because his/her responses followed quite closely the trend found in aggregated data for all the conditions.

Note that high variability in RT does not always correspond to high variability in *ACC*(*RT*) because RT depends on on the speed of the gauge [see Equation (2)]. For example, in Figure 4, when λ = 0.16 and α = 0.75, RTs vary widely across trials, but corresponds to only a slight variation in *ACC*(*RT*).

**Figure 5:**
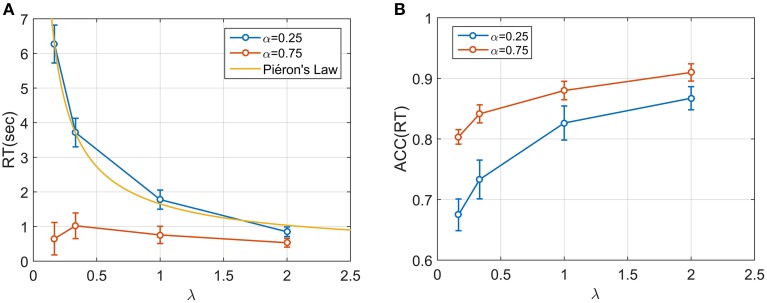
**(A)** Median RT across the 20 participants for four speed conditions and two starting point conditions. A power (Piéron) function was fitted for the low starting point condition (α = 0.25). Median RT appear to be dependent both on λ and α and on their interaction. In particular, when α = 0.75 the mdRT were not monotonic with a peak at λ = 0.33. **(B)** *ACC*(*mdRT*), showing how participant adjusted their positional response along the gauge based on both α and λ.

We next examined median response times mdRT and median positional response *ACC*(*mdRT*), across all (aggregated) participants for each condition (Figures 5A,B). For each participant and condition, the first 10 responses were omitted to avoid contamination from any potential adaptive/learning at the start of a condition. There was a significant effect of both the starting point α and speed λ on mdRT (Two-way repeated measures ANOVA; for λ: $F_{\left(3,\;152\right)}=24.61,\;p<10^{-12}$; for α: $F_{\left(1,\;152\right)}=53.2,\;p<10^{-10}$, and for the interaction α × λ: $F_{\left(3,\;152\right)}=10.84,\;p<10^{-5}$).

For α = 0.25, mdRT decreased monotonically with λ. We were able to capture these data by a power law function $mdRT_{i}=\gamma +k\lambda_{i}^{-\beta }$ for *i* = 1, 4 (yellow line in Figure 5A). The estimated values were γ = 203*ms, k* = 1.45, β = 0.80, with *R*^2^ = 0.997, indicating an excellent fit. This implies an equivalence between λ and conventional stimulus intensity in classic Piéron's law (see Discussion). For α = 0.75, mdRT was much less than for = 0.25, and did not follow a power law. Moreover, mdRT was not monotonic and exhibited a small peak at λ = 0.33.

Similarly *ACC*(*mdRT*) clearly depended on α and λ. This means that participants were actually adjusting their positional response for different conditions and not responding to a fixed position along the gauge independently of λ and α.

### Fitting the models to the aggregate data

**Figure 6:**
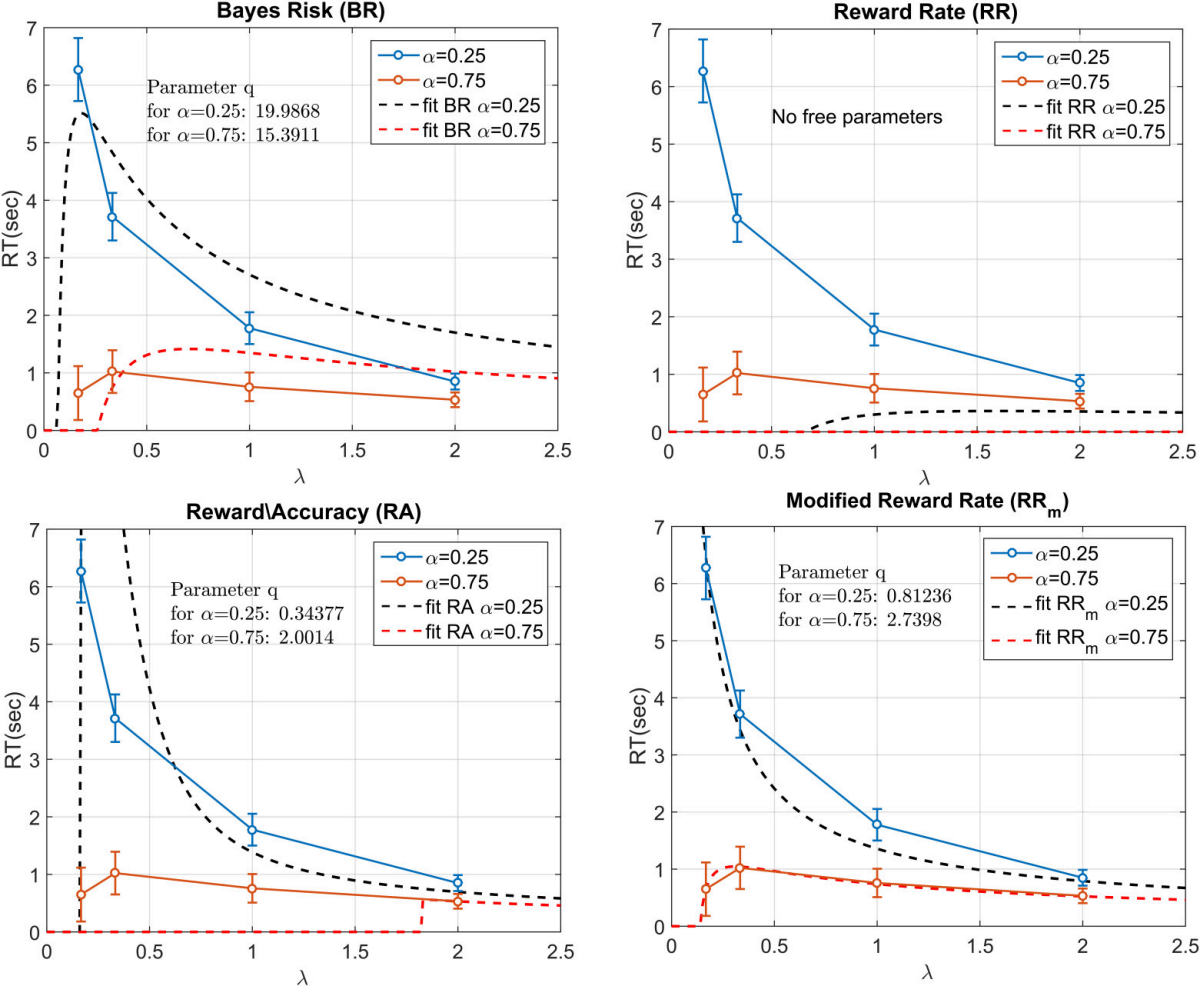
**Fitting the four decision rules to the data by minimizing the least squares**. RR does not have any free parameter, whereas for the other decision rules the free parameter q had to be estimated. In the figure, the parameter *q* is allowed to vary across the two α conditions. The best fit was provided by RR_m_. The two estimated q values were largely different between the two α conditions.

The mdRT were fitted with the optimum t^*^ predictions from four decision rules: Bayes Risk, Reward Rate, Reward/Accuracy, Modified Reward Rate (Figure 6). We minimized the residual least squares by allowing *q* to vary and to be fixed across the two α conditions (this does not apply to RR which does not have free parameters). The plots when *q* is allowed to vary are shown in Figure 6 and the residual least squares in Table 2. When *q* was allowed to vary across α conditions, the only model that could provide a reasonable fit was RR_m_. The estimated *q* (showed in Figure 6) clearly differed from the two α conditions (see Discussion). When the parameter *q* was fixed the resulting curves did not seem to be able to capture the different shape between the two α conditions. In particular the residuals for α = 0.25 were similar for the two fitting methods, but they were higher with fixed *q* in the α = 0.75 condition (see Table 2). Note that with α = 0.75, a residual value of 2.32 corresponds to a horizontal zero line.

**Table 2:** **Residual least squares for each decision rule and the two starting point conditions**.

	**α** = 0.25		**α** = 0.75	
	**q free**	**q fixed**	**q free**	**q fixed**
BR	3.43	3.53	1.08	1.55
RR	55.56		2.32	
RA	36.64	36.64	2.04	2.32
RR_m_	0.29	0.29	0.0009	2.32

*Models were fitted by fixing q across α conditions or by allowing it to vary (this does not apply to RR, which does not have any free parameter). A residual least square value of 2.32 for α = 0.75 corresponds to a horizontal zero line. Fixing q resulted in consistently poor fit with the α = 0.75 conditions. The best fitting decision rule was RR_m_. When q was allowed to vary, RR_m_ provided an excellent fit of empirical data for both the α conditions (see Figure 6)*.

### Time series analysis

**Figure 7:**
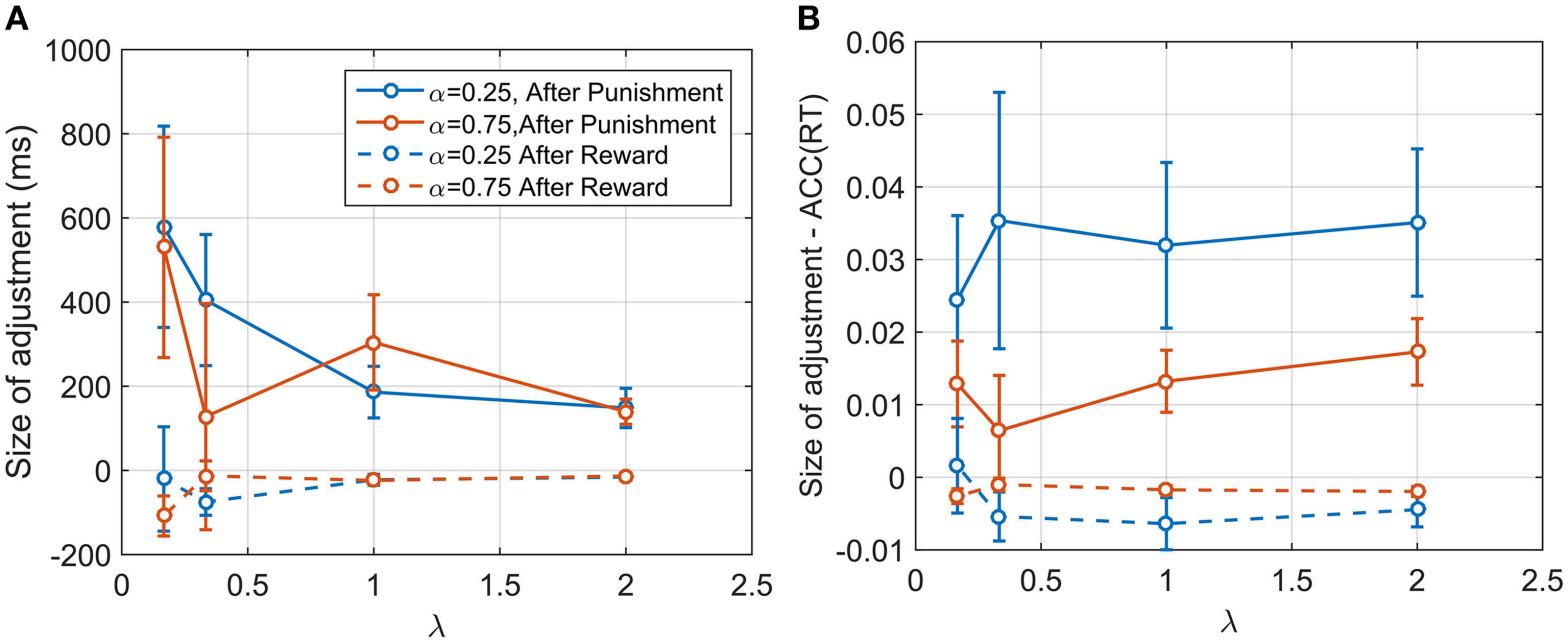
**(A)** Size of the adjustment in ms after a punishment and after a reward for α and λ. The adjustment is larger after a punishment, and seems to be dependent on the conditions itself. **(B)** Size of adjustment in terms of *ACC*(*RT*). As before, the adjustment is larger for punishment. Contrary to what shown in **(A)**, the plot reveals that the size of adjustment is dependent on α and more weakly on λ. It is plausible that participants were adjusting their accuracy by a certain proportion, and in so doing changed RT depending on λ.

We examined sequential dependencies of RT by calculating the partial autocorrelation function (PACF) averaged across participants and conditions (see Materials and Methods). The mean PACF with lag one was 0.457, and with lag two was 0.090. Higher order lags were negligible. Thus, responses depended explicitly on the previous two responses. To explore this further, we examined how this sequential dependency depended on the reward/punishment on previous trials. We computed differences between consecutive RT responses depending on whether the previous response had been punished or rewarded. These differences were averaged across participants and conditions. For a punishment, the mean change in RT was an increase of 345.92 ms (SE: 97.95) and for a reward, a decrease of 61.88 ms (SE: 30.32 ms). For two punishments in a row, the mean increase was 583 ms (SE: 139.26), and for two rewards in a row, the mean decrease was 94.01 ms (SE: 28.62). For three or more rewards/punishments in a row, there was little additional change, consistent with the PACF results. Repeating the analysis for *ACC*(*RT*) showed a similar pattern. After one punishment there was a mean increase of 0.0218 (SE: 0.0058), and after two punishments in a row, a mean increase of 0.048 (SE: 0.0082). After a reward there was a mean decrease 0.0038 (SE: 0.0022), and after two rewards a decrease of 0.0066 (SE: 0.0020). We also analyzed how the 1st order changes depended on λ and α. The size of RT adjustment after a punishment became larger for smaller λ, but with no obvious dependency on α (Figure 7A). However, the change in *ACC*(*RT*) showed the opposite pattern, with strong dependency on α and a weaker dependency on λ (Figure 7B).

The time course of participants' responses during a condition showed variability and small trends. The mean and standard deviation for each participant's response during a condition was computed by dividing the total duration (3 minutes) into bins of 5 or 10 s depending on the condition (see Materials and Methods), and then we performed a linear regression against time. There was little difference between RT and *ACC*(*RT*), so here we consider only RTs. Considering the changing in RT mean across condition duration, we found that the slope was significantly affected by both λ and α conditions (Two-way repeated measure ANOVA; for $\lambda :F_{\left(3,\;152\right)}=10.49,\;p<10^{-13}$, for α:*F*_(1, 152)_ = 12.15, *p* < 0.001). However multiple comparison tests on the slopes of the fitted regression model showed that the only significantly different condition (at 0.01 critical level) was found for α = 0.25 and λ = 0.16, which corresponded to an average increase of 13.5 ms for second (99% CI [9 ms, 17 ms]). None of the other slopes were significantly different from zero. An analysis on the slope of standard deviation of RT vs. time revealed no significant differences from zero for any of the λ and α conditions.

### Distribution analysis

**Figure 8:**
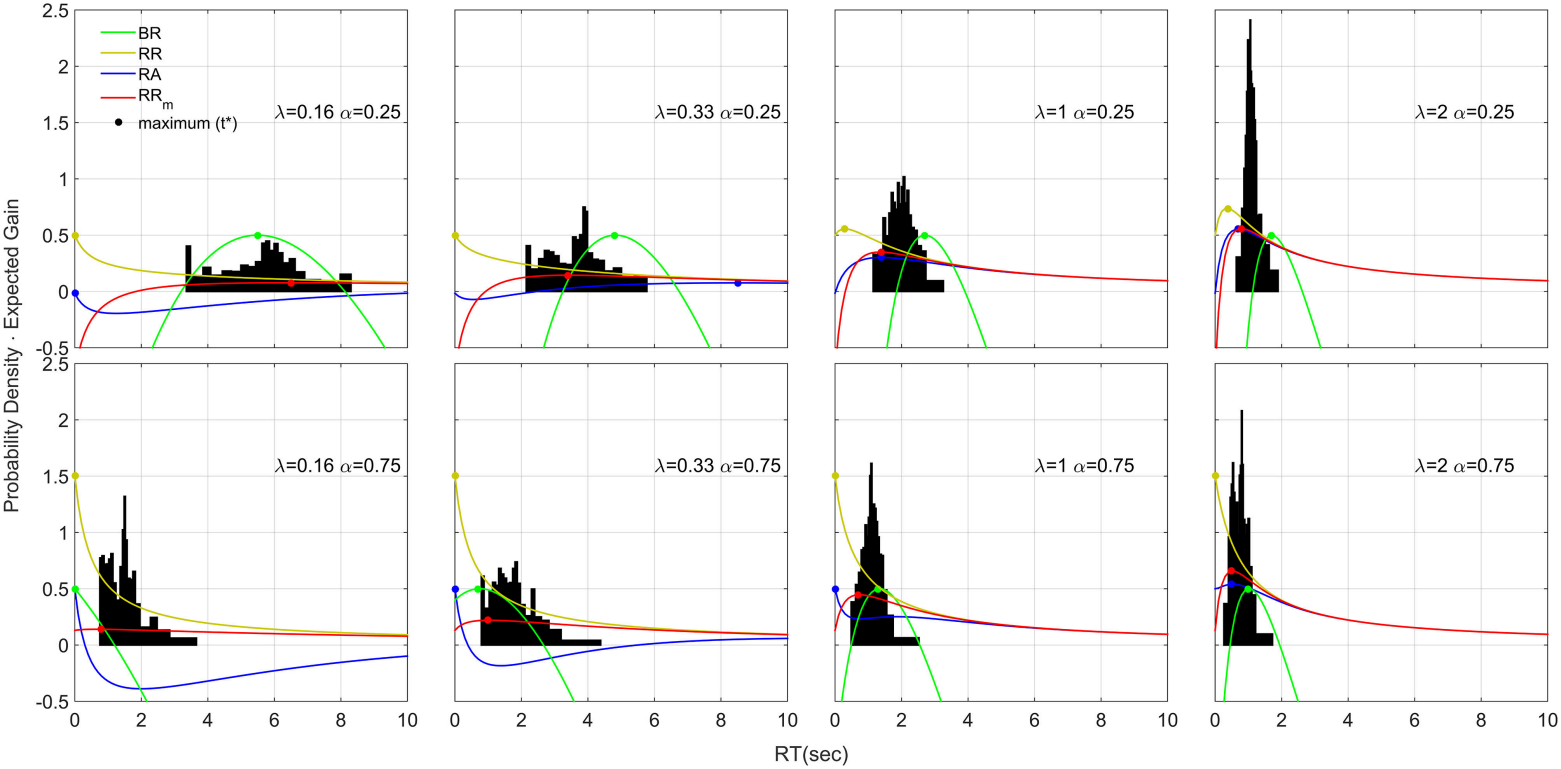
**Response Distributions for each condition obtained by averaging the quantiles across each participant and each condition (Vincentizing)**. The distributions are scaled so that the area of each one is unity. The distributions appear to be skewed on the right side and their shape depends on λ and α. The colored lines refer to four decision rule functions. Note that with respect to the decision rules, the vertical axis corresponds to the expected gain given that particular decision rule. To increase visibility we shift the BR function up by subtracting to the function its maximum value (which was negative) and adding 0.5. In this way the maximum of the BR is always 0.5, but the shape is unchanged. Note that the absolute value of the function is not important for the distribution shape, but the time-depending shape of the decision rule function is. Therefore, shifting the function up or down does not have any theoretical implication for the distribution shape.

The resulting distributions collapsed across participants are shown in Figure 8. The distributions are right skewed (apart from the condition λ = 0.16, α = 0.25). We used the estimated *q* values found with the aggregated data (shown in Figure 6) to plot for each condition the four decision rules functions (colored curves), representing the expected gain for that decision rule (the equation for each decision rule function is shown in Table 1, left column). If a participant is trying to maximize a decision rule, she should always respond precisely at t^*^ (filled circles in Figure 8). With time estimation error, temporal uncertainty, and other source of sensory/motor noise, participants might respond according to a distribution that roughly follows the shape of the decision rule function, with the maximum point slightly ahead of the distribution mean (see Section Asymmetricity of the decision rule and optimization algorithm for further discussion). In our data RR_m_ is the only decision rule that follows this pattern (the only exception being for α = 0.75 and λ = 0.16).

**Figure 9:**
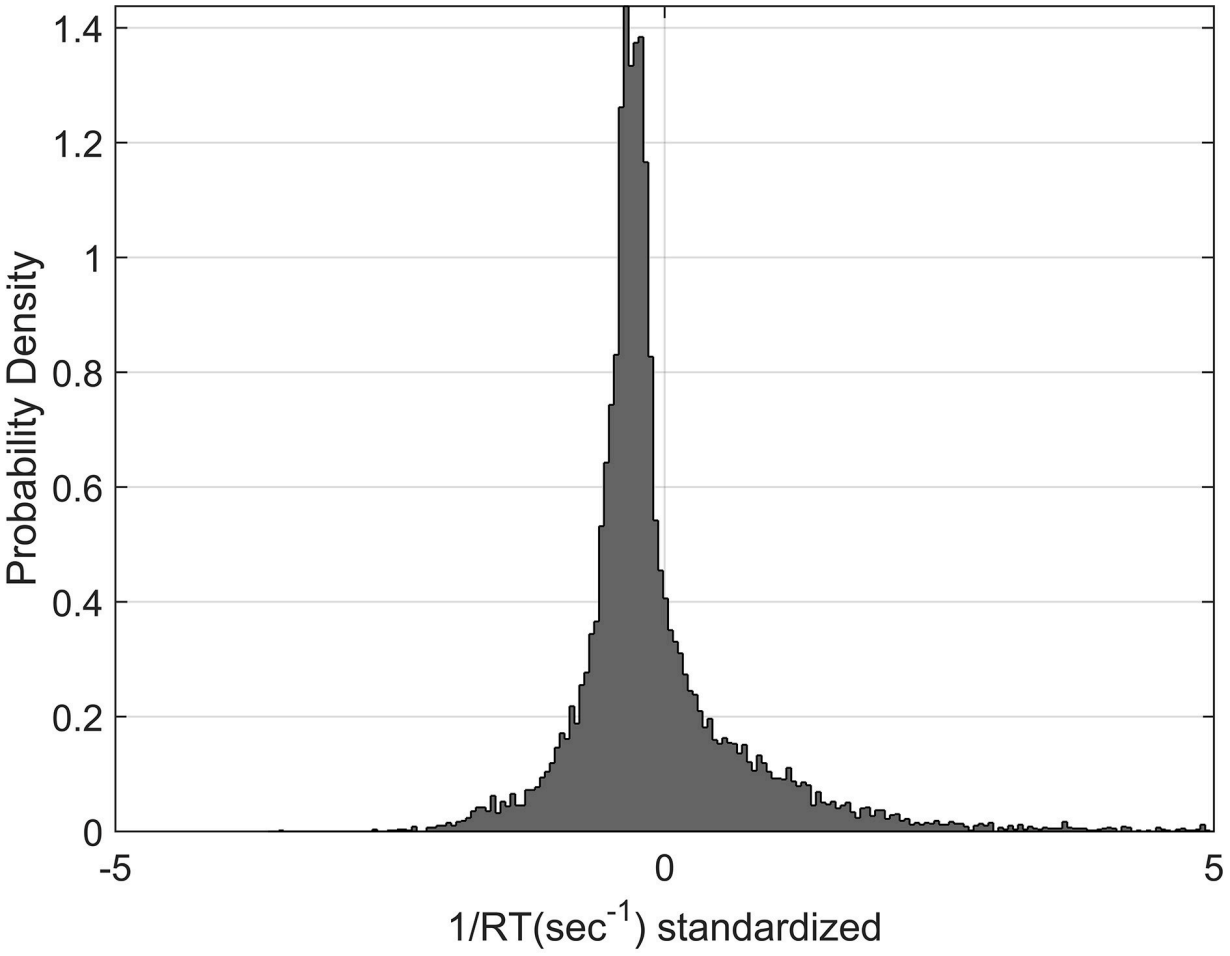
**Standardized Rate Distributions obtained by collapsing the standardized rate for all participants and all conditions (see Materials and Methods)**. The distribution is clearly not normal. Similar shapes were found when analysing each condition separately.

The distribution in the rate domain was analyzed by using the standardization approach (see Materials and Methods). The distributions did not appear normal as observed in choice RT, but were positively skewed. Figure 9 shows the standardized rate distributions collapsed across all the participants and conditions (there was no significant difference between different conditions).

**Figure 10:**
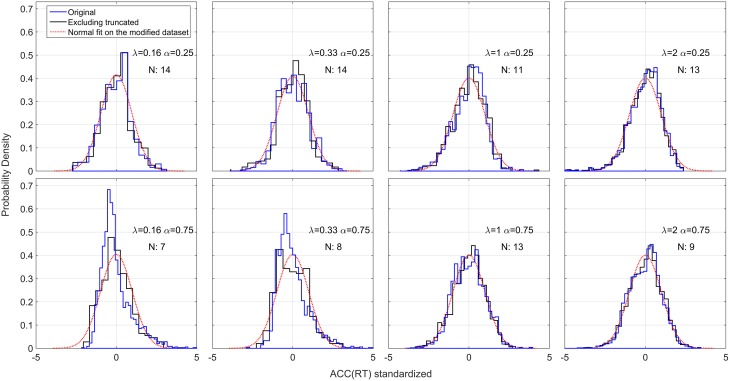
**Standardized positional response along the gauge: *ACC*(*RT*)**. The blue lines represent the original standardized and collapsed distributions; the black lines represent the standardized and collapsed distributions after eliminating the datasets with severe truncation. *N* represents the number of remaining datasets (out of a total of 20 participants) after eliminating severely truncated distributions. A normal density is fitted to each modified distribution.

We also examined the distributions of *ACC*(*RT*). Because of the possible left truncation at 0.25 and 0.75 for the two α conditions, and right truncation at 1 for both conditions, we excluded severely truncated dataset (see Materials and Methods). The remaining 89 out of 160 (20 participants × 2 × 4 conditions) mildly truncated datasets were standardized and collapsed across subjects. Figure 10 shows the original, standardized and aggregated distributions (in blue) and the modified standardized and aggregated distributions (in black). As a comparison, a normal distribution is fitted to the modified dataset. Note that for most of the conditions, excluding the truncated datasets does not have a big effect, and both distribution pre- and post-exclusion are approximately truncated Normal. The two exceptions are the distributions in the α = 0.75 and λ = 0.16, 0.33 conditions, which were skewed and clearly not normal before processing. These two conditions also contained the highest number of severely truncated distributions (only 7 and 8 datasets remaining, see Figure 8). These results are in accord with the hypothesis that accuracy of responses is normally distributed and with most severe truncation with when λ is small and α is high (see Section Accuracy is normally distributed).

## Discussion

Participants' responses to the trials clearly indicated sensitivity to the paradigm. Participants did seem to be attempting to win points, as can be seen in Figure 3. Their median response positions along the gauge depended systematically on the accuracy function *ACC*(*t*) (Figure 5A). When the gauge level moved faster (higher λ), participants' RT decreased, and they selected a position further along the gauge [increasing *ACC*(*RT*)]. This implies that participants were not simply choosing some fixed or idiosyncratic position for all λ and α conditions, which would correspond to a decreasing RT but equal *ACC*(*RT*), but they were actually changing strategy for each λ condition.

Their response also depended on the starting point. When α was lower, participants on average waited longer, and their accuracy decreased. The dependence on λ was strongly dependent on the starting point. Participants slowed their response when α = 0.25 and λ = 0.16 (very slow gauge speed). However, the same did not occur with α = 0.75 and a slow gauge speed. In this condition, most of the participants realized that slowing their response would not correspond to a significant increase of accuracy, and therefore their response was faster.

Comparison of the aggregated data to models supported the RR_m_ decision rule. The fitting with RR_m_, when the parameter *q* was allowed to vary, was better than the other models (Figure 6). For all the models, included RR_m_, fixing *q* resulted in a worse fit. The *q* parameter, when compared with experimental reward and punishment, can be interpreted as a measure of risk attitude. Thus, risk aversion/seeking corresponds to observed *q* being greater/lower than actual punishment/reward ratio (1 in our experiment). In our experiment, estimated values of *q* were quite dissimilar for the two starting point conditions, with significantly higher *q* for the *a* = 0.75 condition. This suggests that participants changed their subjective punishment/reward ratio and become more cautious with increasing α. This is consistent with the “fourfold pattern of risk attitudes” in prospect theory (Tversky and Kahneman, 1992), according to which risk-averse behavior (in our terminology, setting a high *q*) are more common when gains have high probability (like in our α = 0.75 condition). Balci et al. (2011) found that the estimated *q* values for the RR_m_ decision rule decreased with training, to ~0.2. The short session duration in our experiment did not allow us to perform the same analysis, but is in principle possible to with the EXACT paradigm.

There may also be a gender difference in the decision rules in the distribution of parameters. Bogacz et al. (2010) found a clear difference in male and female performance, female responses being less optimal (more risk averse) with respect to the RR strategy. The EXACT paradigm could be similarly (and, we believe, more conveniently) used to investigate whether difference in attitude across gender may be captured by a different estimation of the *q* parameter and hence risk aversion. Unfortunately, this analysis is not possible in the current work due to the severe imbalance between female and male, which reflects the female-male ratio in Psychology classes in our University.

Even though RR_m_ is the decision rule that maximizes the rate of gain for any task with a fixed duration (Harris et al., 2014), it has been found (Bogacz et al., 2010) that for some participants, RA may be a better model than RR_m_. However, Bogacz et al. assumed the drift diffusion model. This assumption constrained the set of predictions generated by the decision rule. For some participants the drift diffusion model may not be a good model of the perceptual process, which may affect the goodness of fit of the decision rule. By using the EXACT paradigm, the current work overcomes this problem.

The EXACT paradigm can be considered as an abstract version of a classic RT task, in which more factors are controlled (more specifically, all the accumulation usually performed by the perceptual process is artificially manipulated by the researcher). We believe it is important to compare this paradigm with the classic RT design. Disentangling the perceptual process from the decision rules mean that is possible to understand which features of the experimental data are due to one or the other part of the decision mechanism. For example, the right-skewness of the distributions that we observed in our dataset is usually deemed to be the result of a rise-to-threshold diffusion process. There is no reason why they should emerge in our paradigm because *ACC*(*t*) is exogenous. However, this does not mean that assuming a rise-to-threshold mechanism is unnecessary in a classic RT task. The *ACC*(*t*) in a perceptual task has to emerge from a process that accumulates information, and a rise-to-threshold model seems plausible. Nevertheless, these distribution features can be explained by an alternative process implying that the rise-to-threshold model may be modified/re-interpreted differently.

The results along conditions for an individual participant (Figure 4) and the size of adjustment after a punishment or a reward (Figure 7) show an important point about the EXACT paradigm: the possibility to analyse the result not only in terms of RT, but also in terms of *ACC*(*RT*). This is not possible in the classic RT paradigm because *ACC*(*RT*) is not known by the experimenter, unless a particular perceptual process is assumed. The analysis of *ACC*(*RT*) gives additional information about the strategy employed by the participants. For example, by analysing the size of adjustment in terms of RT after a punishment/reward, we would have concluded that participants adjusted their response based on the speed of the gauge itself. However analysing the data in terms of *ACC*(*RT*) reveals that participants may be focusing on adjusting the positional response along the gauge, trying to increase or decrease their accuracy at the same proportion for different λ.

The EXACT paradigm has some similarities with the “Beat the Clock” task (Simen et al., 2011), in which a participant earns a reward that is an exponential function of time. If the participant responds after the deadline the reward is zero. In our task, waiting increases the probability of getting a reward, but not the reward itself. Furthermore, in our paradigm participants know perfectly the function shape, as it is shown on the monitor screen, whereas in the “Beat the Clock” they have to infer it. Finally, Simen et al. did not fix the condition's length, which is part of our paradigm. Generally, “Beat the Clock” seems more suited for investigating temporal discrimination, whereas the EXACT paradigm is more suited for analysing decision rules.

### Piéron's law

We found that a power law provided an excellent fit for the mdRT in the α = 0.25 condition: $mdRT_{i}=\gamma +k\lambda_{i}^{-\beta }$. In psychophysics, a similar power law describes the relationship between stimulus intensity and mean RT, (Piéron's Law). During the last century, the law has been reported to match observed data in numerous sensory modalities and experimental paradigms (e.g., brightness detection: Piéron, 1914; color saturation: Stafford et al., 2011; taste signals: Bonnet et al., 1999; simple and choice reaction times: Pins and Bonnet, 1996). Obtaining a Piéron's function with the EXACT paradigm strongly supports the idea that λ corresponds to stimulus intensity in a classic RT paradigm. We estimated these parameters to be γ = 203 ms, β = 0.8, and *k* = 1.45. These values are consistent with the literature for Piéron's law (Luce, 1986). Within this new framework, β can be interpreted as being dependent on the relationship between λ and the physical stimulus intensity.

Recently, it has been proposed that Piéron's Law is a necessary consequence of rise-to-threshold decision making (Stafford and Gurney, 2004; Donkin and Van Maanen, 2014). However, as shown in Harris et al. (2014), the RR_m_ decision rule automatically generates a range of optimum responses which follow a power law. Of our 4 tested rules the RR_m_ is the only decision rule that admits both a Piéron shape and a non-Piéron shape depending on α and *q* (see Figure 2 and Appendix in Supplementary Materials). For the RR_m_ model, a Piéron's function is obtained only when α < *q*∕(*q*+1). In a 2AFC, which is the classic paradigm used in studying with Piéron's law, α = 0.5 which requires that *q*>1; that is, the punishment must have a higher subjective magnitude than the reward for the function to follow Piéron's law. On the other hand, the non-Piéron's shape that we have observed for α = 0.75 requires α>*q*∕(*q*+1). This shape has never been observed in classic RT experiments. However, for a 2AFC experiment (α = 0.5), it would require *q* < 1; that is, punishment would need have a *lower* subjective magnitude than reward. Thus, it is conceivable that the non-Piéron function may emerge in a 2AFC experiment, if the experimenter could augment the subjective reward over the punishment by manipulating the obtained gain or by verbal instruction. We are currently exploring this.

### Time series analysis

The PACF showed that responses were dependent on the previous trials with a lag of one and weakly with a lag of two. We found that the change in response depended on whether the previous trial was rewarded or punished, and was particularly sensitive to punishment. A similar phenomenon is found in classic RT task, where trials following an error are usually substantially slower and more accurate than trial following a correct response (post-error slowing, e.g., Rabbitt, 1966; Laming, 1968; Brewer and Smith, 1984; Jentzsch and Dudschig, 2009; Strozyk and Jentzsch, 2012), and has been interpreted as a strategic adjustment of a response criterion (e.g., Brewer and Smith, 1984; Botvinick et al., 2001; Jentzsch and Leuthold, 2006). It is interesting to note that feedback does not give further accuracy information than the gauge, but may provide information on the rate of subjective reward (RR_m_) which is not easily calculated from *ACC*(*t*). It is possible that post error slowing arises the same mechanism, and may therefore reflect a reward rate maximizing algorithm.

Investigating the relation between the size of the adjustment and λ and α revealed the importance of examining *ACC*(*RT*) as well as RT. RT clearly changed based on λ, but not on α. But *ACC*(*RT*) depended on α but only weakly on λ. It is not possible to know exactly whether participants were adjusting their strategy based on RT or *ACC*(*RT*), but the latter seems more likely. Is it conceivable that participants adjusted their accuracy by a certain proportion, and in so doing changed RT via its dependence on λ (Equation 1). The dependency on α may be explained by noticing that, when α = 0.25, participants' range of adjustment is higher, because the gauge starts on a lower level, and therefore they have a larger range for adjustment than in the α = 0.75 condition.

We also examined responses as a time series in order to explore possible strategies (or algorithms) used by the participants to maximize their subjective gain (e.g., exploration vs. exploitation). However, we found little evidence for a learning algorithm. The only significant trend between mean RT and condition duration was found for the condition α = 0.25 and λ = 0.16. In this case, participants appeared to increase on average their response by 13.5 ms. We were not able to explain this trend, and why none of the other conditions showed a significant trend. Change in standard deviation could underpin a gradual switching from an exploratory to an exploitatory phase, as seen in reinforcement learning. However, we found no significant trends. This may suggest that participants are not using a learning algorithm with adjustable exploration factor, or that 3 minutes are not enough to detect any change in their strategy.

### Normality in the rate domain

We have found that response distribution is not normal as seen in manual choice RT task (Harris et al., 2014) and saccades (Carpenter, 1981). This may be because participants are using a different decision mechanism than in a classic RT task. However, a different explanation can be offered. The reason for normality in the rate domain was explained as fluctuation in the relationship between response time and accuracy across trial, due to sensory noise in the stimulus (Harris et al., 2014). However, in our experiment this noise is drastically reduced, since *ACC*(*t*) is always the same within a block. The response distribution could be a mixture of an optimization algorithm (see next section) and a fluctuation in *ACC*(*t*). In classic RT experiment, the latter may usually mask the optimization algorithm. With the EXACT paradigm, it is possible to design an experiment in which *ACC*(*t*) fluctuates across trials and test for normality in the rate domain, similarly to what predicted by Harris et al. (2014), but has yet to be tested.

### Effect of conditions on distributions

We found that, in all conditions (except with α = 0.25, λ = 0.16), the RT distributions were skewed on the right, similarly to what has been found in classic RT tasks. Also, increased mean resulted in increased the standard deviation of the distribution. These results are both observed in classic RT task (Luce, 1986). Within the framework of diffusion models, these results are explained by taking into account the geometry of the perceptual process. Most rise-to-threshold processes will in fact produce a right-skewed distribution which will generate less spread out distributions with more difficult conditions (higher mean RT; Ratcliff and Rouder, 1998). The explanation in terms of diffusion process does not seem to hold in this paradigm, since the participant is not accumulating information about a particular stimulus within a single trial, as usually assumed. We propose two alternative explanations for these results. Both explanations are for now speculative and clearly require more investigation. They both aim to describe what may be the underlying process that generates the distributions' shape observed without relying on a rise-to-threshold model.

#### Asymmetricity of the decision rule and optimization algorithm

**Figure 11:**
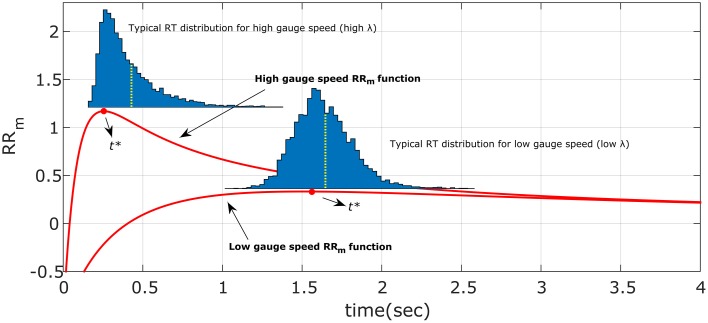
**Illustration of the hypothesized optimum distributions for the RR_m_ decision rule**. The two RR_m_ functions (in red) for two different gauge speeds (different λ) are maximized by responding precisely at t^*^. However, if the response is noisy, the asymmetry of RR_m_ make more profitable in terms of reward rate to err on the right side of t^*^ than on the left. The asymmetry effect is diminished with low gauge speed (small λ): the distributions are less skewed and more spread out. This also entails that the difference between distribution mean (yellow dotted lines) and t^*^ is greater with high gauge speed. Compare these simulated distributions with the empirical data in Figure 8.

The right-skewed distributions and the effect of different λ and α may be due to the way the participants search for the optimum t^*^, and in particular may be due to the asymmetry of the decision rule functions (see Figure 11, red lines, for the RR_m_ decision rule, with a simulated dataset of responses). This might happen because responses that were too fast (*t* < t^*^), would incur more cost than responses that were too slow (*t* > t^*^) (for a given magnitude of error). Thus, any search strategy that was sensitive to the reduction in optimal reward rate would tend to err to the right side of the optimum t^*^. Since the asymmetry of the decision rules depends on λ and α, this could also explain the relationship between distribution shape (e.g., standard deviation and skewness) and experimental condition found in our data (Figure 8). For example, when λ is small (slower gauge speed) or α is small the RR_m_ decision rule function becomes more symmetric and more flat, which implies that participants' gain in terms of RR_m_ would not change much, even with high response variability: the distribution would become less skewed and more spread out (compare the simulated distribution in Figure 11 with our empirical results in Figure 8, upper left panel). On the other hand, as λ increases, the RR_m_ function becomes asymmetric, which would lead to a skewed and less disperse distribution. This distribution would also be biased on the right side of t^*^ (red circles in Figures 8, 11). In our data, most of the distributions follow for the RR_m_ decision rule, but not for the other decision rules. A peculiar exception is found with λ = 0.16 and α = 0.75, but this could be partly due to the maximum point being close to zero, which would force the participant to produce a skewed distribution regardless of the smooth RR_m_ function.

This idea is an extension of an approach already presented by Bogacz et al. (2006) and Balci et al. (2011). In the latter work, empirical thresholds for the drift diffusion model were consistently found to be higher than the optimal threshold, which was deemed to be due to the asymmetry of the RR_m_curve. To our knowledge the same reasoning has not been applied to RT distributions or to the relationship between experimental conditions and distribution shapes.

Taking the asymmetry of the decision rule into account also means to slightly change the predicted t^*^ when fitting the data: all the prediction should be slightly slower than the one used now. We did not consider this, but suggest it as a possible future direction.

#### Accuracy is normally distributed

An alternative explanation could be that participants' responses are actually distributed such that their *accuracy* is normally distributed around *ACC*(t^*^), where t^*^ is the optimum point according to the decision rule used by the participants and the standard deviation can be interpreted as the individual precision parameter. *ACC*(*RT*) cannot be analyzed in classic RT task because the accuracy of each single response is not known, whereas in the EXACT paradigm we can translate RT in *ACC*(*RT*) by Equation (1), and *ACC* in *RT*(*ACC*) by Equation (2).

Note that we should actually consider a truncated normal distribution in the accuracy domain (upper truncation is always one, and lower truncation is α in our experiment and 0.5 in 2AFC). The probability density is found to be equal to

(3) $$\begin{array}{l} RT\left(t;t^{*},\lambda ,\alpha \right)=\\ \frac{\phi \left(1+\left(\alpha -1\right)\exp\left(-\lambda t\right),ACC\left(t^{*}\right),\sigma \right)\left(1-\alpha \right)\lambda \exp\left(-\lambda t\right)}{\Phi \left(1,ACC\left(t^{*}\right),\sigma \right)-\Phi \left(\alpha ,ACC\left(t^{*}\right),\sigma \right)} \end{array}$$

**Figure 12:**
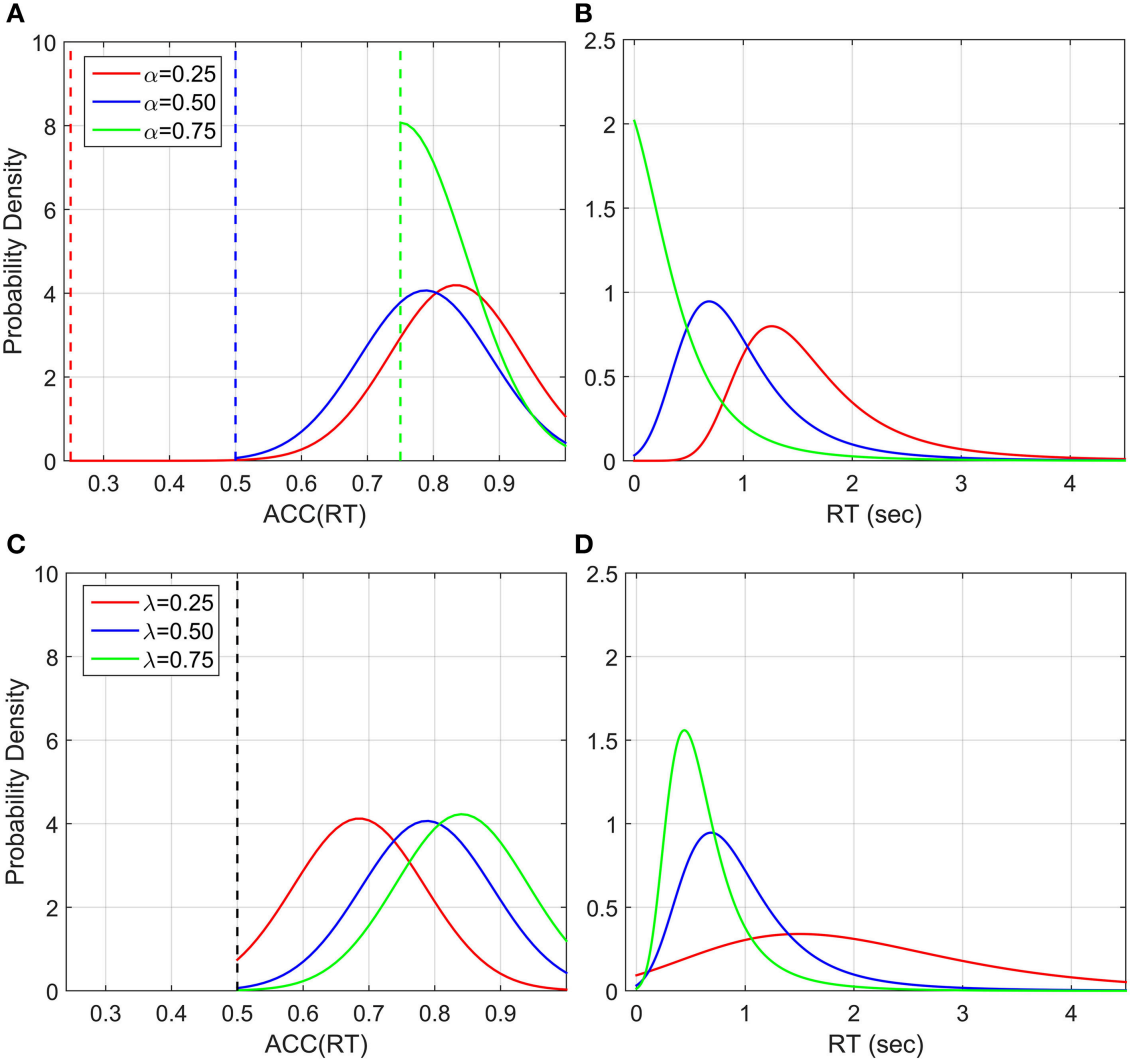
**Illustration of *ACC*(*RT*) distribution and corresponding RT distribution if ACC is normally distributed with truncation at 1 and at various α's**. The mean of *ACC*(*RT*) is $t^{*}{}_{RR_{m}}$ and the standard deviation is set to 0.1 for illustration, which can be interpreted as a precision parameter. **(A)** *ACC*(*RT*) distributions for three α conditions with λ = 1. The dashed vertical lines correspond to the truncation point. **(B)** Corresponding RT distributions. By increasing α, which corresponds to decreasing the number of alternatives in a classic RT task, the distribution is less spread out and the mean decreases (faster responses). **(C)** *ACC*(*RT*) distributions for three λ conditions with α = 0.5 and **(D)** corresponding RT distributions. By increasing λ, which corresponds to change the trial difficult in a classic RT task, the distribution become less spread and the mean decreases (faste responses).

Where ϕ is the normal probability function and **Φ** is the cumulative normal probability function. Figure 12 shows some examples of this distribution with different λ and α parameter. This generates an interesting relationship between the number of alternatives (1∕α), and the trial difficulty (λ). By increasing λ or α the distributions of RT become less spread out and the mean decreases, corresponding to faster responses. This is similar to what we empirically found with this paradigm and what is normally found in the classic RT task (Luce, 1986). This model does not take into account a sensory motor delay, but adding it would not drastically change the predictions.

In our dataset, most accuracy distributions were approximately Normal. However, this was not the case with small λ and high α (see Figure 10). Normality may be masked by severe truncation of the original datasets. In fact, as predicted by our model (Figure 12), the condition with small λ and high α contained the most severe truncated distributions. By excluding the most severely truncated distributions the remaining dataset appear less skewed but the deviation from Normality is still clear. This may be due to the small sample remaining after eliminating the seemly truncated distributions. It would be interesting to test whether accuracy of response distributions follow a near-Normal shape in classic RT tasks.

### Limitations of the exact paradigm

The EXACT paradigm has some limitations and differences from the classic RT task. First, in the EXACT paradigm, the total time between the beginning of the trial and the participant's response corresponds to an increase in reward probability and includes the motor response time. It is not clear if participants take the motor response time into account and decide to respond accordingly. If not, desired accuracy would always be slightly lower than obtained accuracy. On other hand, in the classic RT experiment, most rise-to-threshold models assume motor response time to be an additional component to the accumulation time (Ratcliff and McKoon, 2008). We designed our experiment so that expected RT were of the order of seconds—much longer than typical motor response times so that any overestimation would be minimal. However, this remains to be further explored.

A second point is that the decision rule may differ between the EXACT and classic RT paradigms. Similarities in RT distribution, relationship between distribution shape and experimental condition, and the emerging of a Piéron's shape function lead us to deduce that participants are indeed using the same decision rule across different paradigms. However, the critical test would be to obtain the non-Piéron function in a low punishment classic RT experiment, which we are currently exploring. However, if this turned out not to be the case, it would still be interesting to understand why. Is the decision rule inherently entangled with the perceptual property of the task, or could it be due to other aspects of the task (different time scale, different instructions, etc.)?

The final limitation concerns the incentivisation scheme used. In our experiment we incentivized participants' with a prize for the overall best performance, but other schemes could be used. For example, earned points could be exchanged for money. We do not think this was important in the present experiment, since the amount of points earned/lost was the same for each condition, but it would be important in an experiment with reward/punishment manipulation. It is conceivable that different schemes may result in different decision rules, and future research could explore this possibility. Indeed, the EXACT paradigm may be a useful procedure for isolating the decision rule for different incentivisation schemes.

## Conclusions

The analysis of the decision strategy used by humans has been constrained by the analysis of the perceptual process underlying the decision itself. We designed an experiment, called the EXACT paradigm, which allows us to analyse participants' decision rules and responses based on the accuracy of their response. Instead of relying on a particular model based on a specific type of sampling process, the accumulator that substitutes the perceptual process is exogenously showed to the participant on a computer screen. This design allowed us to know in advance the relationship between response time and accuracy of response, *ACC*(*t*). In this way it was possible to directly compare different decision rules, and found that RR_m_ provided the best fit. We suggest some innovative way to analyse the dataset that can be easily applied to classic RT tasks. Most importantly, we found relevant similarities between our distributions and distributions found in classic RT tasks: the distributions were generally skewed to the right and their shape depended on the trial difficulty. Two different models, one algorithmic and the other based on the accuracy of response, are proposed to explain the distributions shape and their dependency on experimental conditions. Both of these models establish a clear separation from the classic viewpoint of distributions as a result of a rise-to-threshold mechanism.

This new paradigm opens up new ways to explore the human decision-making process that are difficult or impossible using the classic RT paradigm including: exploring the behavior for unusual *ACC*(*t*) functions (e.g., not-increasing, non-monotonic), easily manipulation of the number of alternatives (α), also by mimicking unusual setups (α>0.5), exploring subjective pay-off (*q*), and exploring the “algorithm” used by participants use to find the optimum response time (t^*^).

### Conflict of interest statement

The authors declare that the research was conducted in the absence of any commercial or financial relationships that could be construed as a potential conflict of interest.

## Footnotes

^1^For example, consider the *ACC*(*t*) resulting from a drift diffusion model with two boundaries (that is, two alternative forced choice task): $ACC(t)=1-\phi \left(-\frac{A}{c}\sqrt{t}\right)$, where A is the drift rate, c is the standard deviation of the process and ϕ is the normal standard cumulative function (Bogacz et al., 2006). Notice that this function is monotonically increasing, concave, and asymptotically approaches unity, similarly to our function. However, the function does not allow us to model different starting points, because the drift diffusion model can be only used in case of two alternative forced choice tasks, and the difficulty of the trial is controlled by two parameters instead of one of our function.

^2^To show why this is true, note that *c*_1_*ACC*(*t*) − *c*_2_(1 − *ACC*(*t*)) (the numerator in RR_m_) is the average gain of a subject for a trial, where *c*_1_ and *c*_2_ are the subjective utility (reward and punishment). For an experiment lasting *l* min, the subject will be able to perform *n* = *l*∕(*t*+*d*) trials, where *t* is the response time of the subject, *d* the other delay of the trial. Therefore to get as many points as possible for an experiment, the participants should maximize $\left[c_{1}ACC\left(t\right)-c_{2}\left(1-ACC\left(t\right)\right)\right]\;\frac{l}{t+d}$, which maximum correspond exactly to $t_{RR_{m}}^{*}$, with *q* = *c*_2_∕*c*_1_.
