# Supplementary material for: Investigating decision rules with a new experimental design: the EXACT paradigm
Source: Front Behav Neurosci. 2015 Nov 3;9:288. doi: 10.3389/fnbeh.2015.00288 (PMC4630306; doi:10.3389/fnbeh.2015.00288)
Supplement: Supplementary file 1 [file DataSheet1.DOCX]

**Appendix**

**Derivation of Optimum Response** $\mathbf{t}^{\mathbf{*}}$ **for the four decision rules**

For each decision rule, we describe the steps to derive the corresponding $t^{*}$ for the exponential speed-accuracy trade-off function: $\mathrm{ACC}\left( t \right)=1+\left( \alpha-1 \right)exp(-\lambda t)$. To find the maximum point in each decision rule, we set the derivative with respect to $t$ equal to 0 and solve for $t.$

For $BR=-\left[ t+q\left( 1-ACC\left( t \right) \right) \right]$, the derivative is

|  | $\frac{\partial BR}{\partial t}=-1-\lambda q\exp\left( -\lambda t \right)\left( \alpha-1 \right)=0$ | (A1) |
| --- | --- | --- |

and hence:

|  | $t_{BR}^{*}= -\frac{\ln\left( \frac{1}{\lambda q\left( 1-\alpha\right)} \right)}{\lambda}.$ | (A2) |
| --- | --- | --- |

Applying L’Hospital’s rule, we found that when $\lambda\to0$ then $t_{BR}^{*}\to0$ and when $\lambda\to\infty$ then $t_{BR}^{*}\to0$.

For $RR=ACC(t)/(t+d)$ the derivative is

|  | $\frac{\partial RR}{\partial t}=-\frac{1-exp \left( -\lambda t \right)\left( \alpha-1 \right)}{\left( t+d \right)^{2}}-\frac{\lambda\exp\left( -\lambda t \right)\left( \alpha-1 \right)}{t+d}=0$ | (A3) |
| --- | --- | --- |

Rearranging:

|  | $\left( 1+\lambda t+\lambda d \right)\exp\left( -\lambda t \right)=-\frac{1}{\alpha-1}$ | (A4) |
| --- | --- | --- |

and setting :

|  | $x=-\lambda t-\lambda d-1$ | (A5) |
| --- | --- | --- |
|  |  |  |

we obtain $x\exp\left( x+\lambda d+1 \right)=\frac{1}{\alpha-1}$ or

|  | $x\exp\left( x \right)=\frac{\exp\left( -\lambda d-1 \right)}{\alpha-1}$ | (A6) |
| --- | --- | --- |

We can now express this relationship in terms of $Lambert W function$, which is defined as $W\left( z \right)\exp[W(z)]=z$. Therefore

|  | $x=W\left( \exp\frac{\left( -\lambda d-1 \right)}{\alpha-1} \right)$ | (A7) |
| --- | --- | --- |

which we can substitute back to (A5) to obtain

|  | $t_{RR}^{*}=-d-\frac{W\left( \frac{\exp\left( -\lambda d-1 \right)}{\alpha-1} \right)+1}{\lambda}$ | (A8) |
| --- | --- | --- |

As the $Lambert W$ function is a multivalued function, we need establish which branch we are referring to. Note that to obtain $t_{\mathrm{RR}}^{*}\geq0$ we need $W\left( \cdot\right)\leq-1-\lambda d$. Since $\lambda,d\geq0$, then $W\left( \cdot\right)\leq-1$ and therefore the function is limited to its lower branch, $W_{-1}$. With $\lambda\to0$ RR become $\frac{\lambda}{t+d}$ and the maximum is reached when $t=0$.

We were not able to obtain an explicit form for RA, not even in terms of Lambert W.

For $RR_{m}=\frac{ACC\left( t \right)-q\left( 1-ACC\left( t \right) \right)}{t+d}$ the derivative is equal to

|  | $\frac{\partial RR_{m}}{\partial t}=-\frac{\exp\left( -\lambda t \right)\left( \alpha-1 \right)+q\exp\left( -\lambda t \right)\left( \alpha-1 \right)+1}{\left( t+d \right)^{2}}-\frac{\lambda\exp\left( -\lambda t \right)\left( \alpha-1 \right)+\lambda q\exp\left( -\lambda t \right)\left( \alpha-1 \right)}{t+d}$ | (A9) |
| --- | --- | --- |

Which maximum $t^{*}$ can be found by setting this derivative equal to zero. By rearranging terms we obtain:

|  | $\left( \lambda t+\lambda d+1 \right) exp(-\lambda t)=-\frac{1}{\left( \alpha-1 \right)\left( q+1 \right)}$ | (A10) |
| --- | --- | --- |

As before, we set $x=-\lambda t-\lambda d-1$, so that we can express $x$in terms of $Lambert W$ function:

|  | $x=W\left( \frac{\exp\left( -\lambda d-1 \right)}{\left( \alpha-1 \right)\left( q+1 \right)} \right)$ | (A11) |
| --- | --- | --- |

Leading to

|  | $t_{RR_{m}}^{*}=-d-\frac{W\left( \frac{\exp\left( -\lambda d-1 \right)}{(\alpha-1)(q+1)} \right)+1}{\lambda}$ | (A12) |
| --- | --- | --- |

For the same argument as before, we know that for $t_{RR_{m}}^{*}$ to be positive the function $W$ is limited to its lower branch, $W_{-1}$. When $\lambda\to0$ $RR_{m}$ become $\frac{\alpha\left( 1+q \right)-q}{t+d}$ which maximum depends on the sign of the numerator. It is $t_{RR_{m}}^{*}=0$ when $\alpha>\frac{q}{q+1}$, $t_{RR_{m}}^{*}=\infty$ when $\alpha<\frac{q}{q+1}$, undetermined when $\alpha=\frac{q}{q+1}$.
